# Repurposing mebendazole against triple-negative breast cancer leptomeningeal disease

**DOI:** 10.21203/rs.3.rs-3915392/v1

**Published:** 2024-02-05

**Authors:** Adrian Rodrigues, Sophia B. Chernikova, Yuelong Wang, Thy T. H. Trinh, David E. Solow-Cordero, Ludmila Alexandrova, Kerriann M. Casey, Elizabeth Alli, Abhishek Aggarwal, Tyler Quill, Ashley Koegel, Brian J. Feldman, James M. Ford, Melanie Hayden-Gephart

**Affiliations:** Massachusetts General Hospital; Stanford University; Department of Neurosurgery, West China Hospital, Sichuan University, Chengdu, China; Department of Neurosurgery, Stanford School of Medicine, Stanford, CA 94305; High-Throughput Screening Knowledge Center, Sarafan ChEM-H, Stanford CA 94305; Vincent Coates Foundation Mass Spectrometry Laboratory, Stanford University, Stanford, CA, 94305; Department of Comparative Medicine, Stanford University School of Medicine, Stanford, CA 94305; Department of Cancer Biology, Wake Forest University School of Medicine, Winston-Salem, NC 27109; High-Throughput Screening Knowledge Center, Sarafan ChEM-H, Stanford CA 94305; Department of Materials Science and Engineering, Stanford University, Stanford, CA 94305; Department of Pediatric Hematology-Oncology, University of California, San Francisco, CA 94143; Department of Pediatrics, University of California, San Francisco, CA 94143; Department of Medicine (Oncology), Stanford School of Medicine, Stanford, CA 94305; Department of Neurosurgery, Stanford School of Medicine, Stanford, CA 94305

**Keywords:** Breast cancer, leptomeningeal disease, drug repurposing, mebendazole

## Abstract

**Purpose:**

Triple-negative breast cancer (TNBC) is an aggressive subtype that often metastasizes to the brain. Leptomeningeal disease (LMD), a devastating brain metastasis common in TNBC, has limited treatment options. We sought to test whether the common anti-helminthic drug mebendazole (MBZ) may be effective against murine TNBC LMD.

**Methods:**

A small-molecule screen involving TNBC cell lines identified benzimidazoles as potential therapeutic agents for further study. *In vitro* migration assays were used to evaluate cell migration capacity and the effect of MBZ. For *in vivo* testing, LMD was introduced into BALB/c athymic nude mice through internal carotid artery injections of brain-tropic MDA-MB-231-BR or MCF7-BR cells. Tumor growth and spread was monitored by bioluminescence imaging. MBZ was given orally at 50 and 100 mg/kg doses. MBZ bioavailability was assayed by mass spectrometry.

**Results:**

Bioinformatic analysis and migration assays revealed higher migratory capacity of TNBC compared to other breast cancer subtypes. MBZ effectively slowed down migration of TNBC cell line MDA-MB-231 and its brain tropic derivative MDA-MB-231-BR. In animal studies, MBZ reduced tumor growth and extended survival in the LMD model produced by MDA-MB-231-BR cells. MBZ did not have an effect in the non-migratory MCF7-BR model.

**Conclusions:**

We demonstrated that MBZ is a safe and effective oral agent in an animal model of TNBC LMD. Our findings are concordant with previous efforts involving MBZ and central nervous system pathology and further support the drug’s potential utility as an alternative therapeutic for TNBC LMD.

## Introduction

Triple-negative breast cancer (TNBC) is an aggressive subtype that metastasizes to the brain in up to 50% of affected patients [1]. Once disseminated to the central nervous system (CNS), TNBC carries a poor prognosis, with limited treatment options [2] and a median survival of only 5 months [1]. Leptomeningeal disease (LMD, also known as leptomeningeal carcinomatosis, neoplastic meningitis, or carcinomatous meningitis) is the metastatic spread of cancer to the pia mater, arachnoid mater, and subarachnoid space [3]. LMD is characterized by fast progression: once in cerebral spinal fluid (CSF), cancer cells disperse widely along the surface of the brain and spinal cord. While LMD is documented to occur in a minority of breast cancers, its incidence is increasing [4, 5]. Among breast cancer subtypes, TNBC accounts for the shortest time between primary diagnosis and CNS metastasis and has the highest propensity to metastasize to the brain and develop LMD [6]. Rapid metastatic dissemination of TNBC is likely based on its high migratory potential [7]. Current standard-of-care for LMD involves systemic and intrathecal chemotherapy with- or without palliative whole brain radiation, with limited efficacy [8].

Given the paucity of effective treatments and challenges with identifying and approving new drugs for relatively small patient populations, recent work has begun to focus on repurposing previously approved pharmaceutical agents. This approach has two main advantages over the de *novo* development: substantially reduced costs and an accelerated time-to-patient use [9]. In the field of neuro-oncology, drug repurposing is of particular interest due to the high cost of new therapies and the limited effectiveness of available treatments [3, 4, 8].

In the study of CNS tumors, drug repurposing efforts have highlighted the benzimidazole anti-helminthic class, including mebendazole (MBZ), albendazole (ABZ), and fenbendazole (FBZ) [10, 11]. This drug class is widely used in the control of human and animal parasitic infections by disrupting microtubule function, and via this mechanism, it has demonstrated efficacy against paclitaxel and doxorubicin-resistant cancer cells [12], glioma and metastatic TNBC [10, 13, 14]. MBZ and ABZ have been used against CNS pathologies (e.g., neurocysticercosis and cerebral echinococcosis) [15–17], indicating sufficient CNS bioavailability. This has allowed for the fast transition to clinical testing of MBZ in adult high-grade glioma and pediatric glioma [18–20].

The effectiveness of MBZ in animal models of glioma and metastatic TNBC highlight both its potential as an alternative oncologic therapeutic and its potential utility against LMD, another pathology of the CNS. *In vivo* experimentation of drug repurposing in LMD models has not yet been conducted. The identification of promising agents in preclinical LMD models would represent substantial progress in the investigation and treatment of this condition. In the present study, we hypothesized that the tubulin-binding properties of MBZ would counter the migratory capacity of TNBC LMD and, therefore, delay mortality.

## Methods

### Cell culture

Brain-tropic luminal A MCF7-BR cell line was established in our laboratory from MCF7 cells through two cycles of internal carotid (ICA) injection, selection from brain, and *in vitro* propagation. Brain-tropic TNBC MDA-MB-231-BR3 cell line, a kind gift from Dr. J.E. Price (M.D. Anderson Cancer Center, Houston, TX), was obtained from brain metastases formed after ICA injection of MDA-MB-231 cells, as described [21]. All cell lines were maintained in Dulbecco’s Modified Eagle Medium (DMEM) supplemented with 10% fetal bovine serum and antibiotics, and incubated at 37°C in humidified air containing 5% CO_2_. The MDA-MB-231-BR and MCF7-BR cell lines were transfected with firefly luciferase to enable later *in vivo* luminescence imaging.

### High-throughput cytotoxicity assay

The high-throughput screen of the Sum149PT and MCF10a cell lines was conducted as previously described [22]. The viability of MDA-MB-231 and MCF7 cell lines was determined 24 hours and 48 hours after compound addition, respectively. Cell-Titer Blue assay and Bright-Glow luciferase assay (Promega, WI) were used to assess cell viability. For all assays, the compounds were tested in a 7-point dose response at a final concentration of 20, 10, 5, 2.5, 1.25, 0.625, and 0.3125 μM. We tested the following libraries: LOPAC1280, Microsource Spectrum (2000 compounds), and the Biomol ICCB bioactive (480 compounds) and FDA-approved library (640 compounds).

### Cell viability assay

Cells were seeded at 500–1000 cells/well in 96-well tissue culture plates. MBZ was added to the cells after 16 hours incubation at 37°C. The MTS assay (CellTiter Aqueous One Assay, Promega, WI) was performed on day 7 following drug addition.

### Migration assay

Cell migration was evaluated using transwell migration assay (6.5 mm diameter inserts with 8 μm pore size, polycarbonate membrane (Costar 3422, Corning)). Cells resuspended in serum-free media at a density of 500,000/ml were pretreated with various concentrations of MBZ and 200 μl of cell suspension was added to a top chamber of a 24 well plate. Cells were allowed to settle for 10 minutes, placed into a lower chamber containing 500 μL of complete media (DMEM-10% FBS) and incubated at 37°C. At the end of each incubation period, cells were washed twice by gently dipping chamber into a beaker with cold PBS, fixed with 100% methanol for 10 min at −20°C and stained with crystal violet (0.5% crystal violet in 25% methanol/PBS) at room temperature for 10–15min. Stain was removed by dipping the chamber in tap water until dye stopped coming off. The membranes were counterstained with 0.3 μM DAPI and rinsed in PBS. Non-migrated cells in the top chamber were rubbed off with a cotton swab stick, making sure that all cells from the edge of the membrane in the top chamber were removed. The membrane was allowed to dry, carefully excised from the well, mounted on a microscope slide, and imaged. Alternatively, the membrane was incubated in 500 μl of 10% acetic acid to dissolve stain with shaking for 10–15 min at room temperature. 150 μL was transferred to a 96 well plate and read OD at 570 nM (Fig. S1).

### Mass spectroscopy detection of MBZ in CSF

Individual analyte and internal standard primary stock solutions (10 mM) were prepared in DMSO. Intermediate stock solutions of MBZ and ABZ were prepared separately in acetonitrile/water (1:1 v/v) buffer. MBZ intermediate stock solution was serially diluted with acetonitrile/water (1:1 v/v) buffer to obtain standard working solutions to generate calibration curves. Calibration curves were prepared by spiking 10 μL of each of the standard working solutions into 50 μL of blank mouse plasma or into 10 μL artificial CSF followed by the addition of 10 μL internal standard solution of ABZ (250 ng/mL for plasma analysis and 25 ng/mL for CSF analysis). Calibration curves were prepared fresh with each set of samples. Calibration curve ranges for MBZ were 4 to 4000 ng/mL for plasma and 0.5 to 500 ng/mL for CSF.

50 μL aliquots of plasma or 10 μL aliquots of CSF were used for analysis. 10 μL internal standard solution was added to 50 μL plasma (or 10 μL CSF) aliquot and mixed by vortexing. 200 μL ice cold solution of methanol/1% acetic acid was added to the sample, samples were vortexed and incubated 1 hour at −20°C to facilitate protein precipitation. After centrifugation, 50 μL (plasma) or 40 μL (CSF) of supernatant was transferred to a new vial, diluted with 25 μL (plasma) or 20 μL (CSF) water, and analyzed by LC-MS/MS.

All analyses were carried out by positive electrospray LC-MS/MS using a Waters Acquity I-class UPLC system with Waters Xevo TQ-XS triple quadrupole mass spectrometer (RRID:SCR_018510). Chromatographic conditions: a Acquity UPLC^®^ BEH C18 2.1×50 mm 1.7 μm particle size column (Waters Corp., part number 186002352) was operated at 40°C at a flow rate 0.25 mL/min. Mobile phases consisted of A: 0.2% formic acid in water and B: 0.2% formic acid in acetonitrile. Elution profile: initial hold at 25% B for 3 minutes, followed by a linear gradient of 25%-98% in 3 minutes, hold at 98% for 1 minute, equilibrate back to 25% B; total run time was 7 minutes. Injection volume was 10 μL. Quantitative analysis was done with TargetLynx quantification software (Waters Corp.) using an internal standard approach.

### Infra-red spectra identification of MBZ polymorphs

MBZ was procured from Sigma Aldrich (catalog #M2523, CAS # 31431–39-7). Infrared spectra were measured using a Nicolet iS50 FT/IR spectrometer (Thermo Fisher, MA) using an attenuated total reflectance (ATR) accessory equipped with a diamond ATR crystal.

### Animal model and tumor implantation

All animal studies were approved by the Administrative Panel on Laboratory Animal Care of Stanford University. Unilateral ICA injections were performed in female NuNu mice (Charles River Laboratories) as previously described [23]. Cells were injected in a volume of 20 μl: MDA-MB-231-BR (20,000 cells) and MCF7-BR (50,000 cells). To prevent cell reflux, both the ipsilateral external carotid artery and ipsilateral common carotid artery were ligated. The ICA injection method has also been used previously to model the spread of helminthic cysts [24].

The mice were randomly divided into treatment and control groups once tumor size exceeded 2.5 × 10^5^ photons/sec on bioluminescence imaging (BLI). MBZ was given daily at 50 or 100 mg/kg as oral voluntary ingestion as previously described [25]. These doses have been shown to be effective in murine models of glioma [11, 13, 26]. MBZ emulsion in pure sesame oil was diluted 1:1 with honey and the resulting suspension was diluted 1:1 with 1% hydroxycellulose. Sesame oil was used to augment MBZ enteral absorption [26], and hydroxycellulose was used to prevent MBZ precipitation. Honey increased animal motivation to voluntarily eat the suspension [25]. Control animals received a suspension of hydroxycellulose, pure sesame oil and raw honey. The animals were treated with oral MBZ or control solution daily for the first 21 days and then every 48 hours thereafter. Twice-weekly BLI provided quantitative *in vivo* approximates of tumor size, and Kaplan-Meier curves assessed differences in survival. Mice were monitored daily for signs of drug toxicity. For further immunohistochemistry analyses brain tissues were perfused with PBS, dissected and frozen in Optical Cutting Tissue embedding medium.

### Immunohistochemistry

Frozen sections (10 μm) were dried, fixed in 4% paraformaldehyde, quenched in 50 mM NH4Cl and permeabilized by 0.5% Triton X-100. MDA-MB-231-BR3 cancer cells were detected using antibodies against human vimentin (Millipore, CBL202) followed by a secondary Alexa Fluor 488 anti-mouse Fc-gamma subclass 2a specific antibody (Jackson ImmunoResearch Labs, 115–545-206). The following secondary antibodies were used in other applications - Alexa Fluor 488, Alexa Fluor 594, Alexa Fluor 568 (Molecular Probes). MCF7 cells were detected by anti-Pan-cytokeratin antibody (Novus Biologicals, NBP2–33200) or mouse monoclonal anti-human estrogen receptor alpha antibody (Santa Cruz Biotechnology, sc-8002-AF594). Endothelial cells were detected using rat anti-mouse CD31 (BD Pharmingen, 550274) or rat anti-mouse PV1 (BioRad, MCA2539T) antibodies. Cell nuclei were detected with DAPI. Whole skulls were fixed for 72 hours using a combined fixation and decalcification protocol (Cal-Ex II, Fisher Scientific, CS511–1D), sectioned and stained with hematoxylin and eosin.

## Results

### Benzimidazoles identified as a potential treatment for LMD

**Figure 1:**
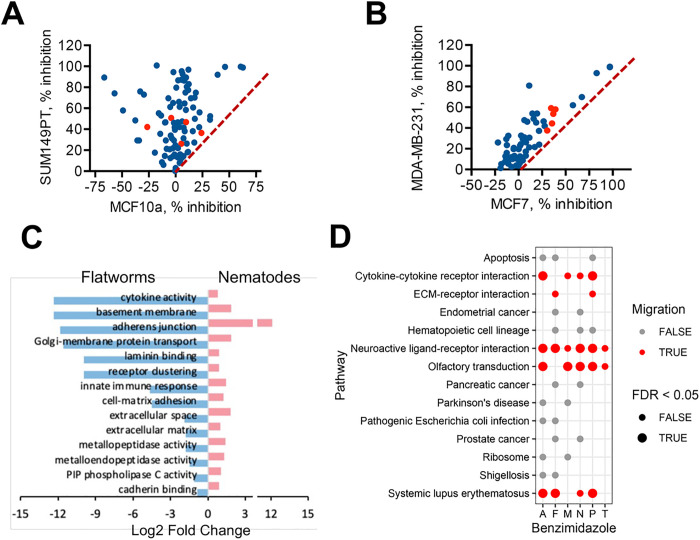
Benzimidazoles as a potential treatment for migratory cancers, such as TNBC. (A) Small molecule screen used to identify pharmacologic compounds active against triple-negative breast cancer (TNBC) cell line SUM149PT, but not against a non-tumorigenic breast cell line MCF10a [22]. (B) Benzimidazoles may be more selective against TNBC. Shown are compounds more effective against metastatic TNBC cell line MDA-MD-231 compared to a metastatic luminal A breast cancer cell line MCF7. (A, B) Diagonal line is placed for agents equally effective against indicated cell lines. Benzimidazoles are labeled in red. (C) GO enrichment terms associated with migration are overrepresented in nematodes and underrepresented in flatworms [32]. (D) Disruption of ligand-receptor interactions important for cell migration and LMD represent a common mode of benzimidazole action in mammalian cells. Results of the Drug-Path database query [35], which shows the pathways significantly affected in mammalian cells by benzimidazoles. The pathways strongly associated with cell migration (in red color) were affected by the majority of tested benzimidazoles [albendazole (A), fenbendazole (F), mebendazole (M), nocodazole (N), parmendazole (N), and thiabendazole (T)] and have a low false discovery rate (FDR).

Initial small molecule screen performed as part of a high-throughput screen [22] revealed that several benzimidazole drugs selectively inhibited growth of a TNBC cell line, SUM149PT, in comparison to a non-tumorigenic epithelial breast cell line, MCF10a (Fig. 1A). In addition, this pharmacologic class exhibited greater inhibition of growth of another TNBC cell line, MDA-MB-231, compared to a luminal A breast cancer cell line MCF7 (Fig. 1B).

The mechanism of the anti-helminthic action of benzimidazoles is believed to be based on their high affinity to parasites’ tubulin [27]. This conclusion is supported by several key observations: 1) selective binding of benzimidazoles to helminths’ tubulin has been found to correlate with high efficacy towards helminths and little toxicity towards mammalian cells [27–29]; 2) the instances of acquired resistance to benzimidazoles were linked to mutations in β-tubulin [30]; and 3) observed much higher efficacy of benzimidazoles against parasitic roundworms (Phylum Nematoda) over that of the flatworms (Phylum Platyhelminthes) was found to correlate with higher affinify to the tubuin of roundworms compared to flatworms [27] (Table S1).

According to the tubulin code theory [31], specific tubulin isoforms and modifications control specific cellular functions. Tubulin of nematodes has been shown to be different from the mammalian tubulin [31]. Therefore, by selective binding to nematode tubulin, benzimidazoles might interfere with tubulin-dependent processes crucial for nematode survival without affecting survival of mammalian host [27–29]. To find which tubulin-dependent processes might be associated with higher sensitivity to benzimidazoles in nematodes compared to flatworms, we querried the gene expression dataset containing data on 56 nematodes and 25 flatworms [32]. Our analysis revealed that Gene Ontology (GO) enrichment terms associated with migration within extracellular matrix (ECM), such as cytokine activity, basement membrane, adhesion, ECM, etc., were overrepresented in nematodes and underrepresented in flatworms (Fig. 1C). These GO terms are known to be associated with invasion and penetration of ECM or other tissue barriers during metastatic dissemination [33]. Of note, the free-living nematode *Caenorhabditis elegans* that has been widely used to model parasitic nematodes, is now being used as an *in vivo* model for the invadopodia-mediated metastatic cancer invasion through the basement membrane [34].

A subsequent query of the Drug-Path database (http://www.cuilab.cn/drugpath), which contains information on gene expression networks affected by various drugs in mammalian cells [35], revealed that in mammalian cells benzimidazoles target pathways associated with cell migration. The biological pathways affected by the six benzimidazoles found in the database (albendazole, fenbendazole, mebendazole, nocodazole, parbendazole, and thiabendazole) converged into common pathways of cell receptor-ligand interactions (Fig. 1D, Table S2), and included interactions with cytokines and ECM. Since these are the same pathways as used by migrating cells of the developing larva, there is a possibility that both in the nematodes’ larvae and in metastatic cancer cells, benzimidazoles inhibit the same processes necessary for cell migration.

Given that benzimidazoles target migration-associated pathways, have sufficient CNS bioavailability, and are active against TNBC, we decided to investigate the efficacy of benzimidazoles in our previously described animal model of TNBC LMD [23]. LMD is characterized by widespread involvement across the CNS: as demonstrated both in rodent models of LMD and in LMD patients, TNBC cells easily cross various basement membranes of the brain, extravasating out of blood vessels and shuttling between leptomeningeal and parenchymal anatomical compartments [36, 37]. As few treatment options are currently available for this aggressive CNS metastasis, the LMD model represented an ideal choice to test the *in vivo* effect of benzimidazoles.

### Mebendazole (MBZ) as a potential treatment against TNBC LMD

**Figure 2:**
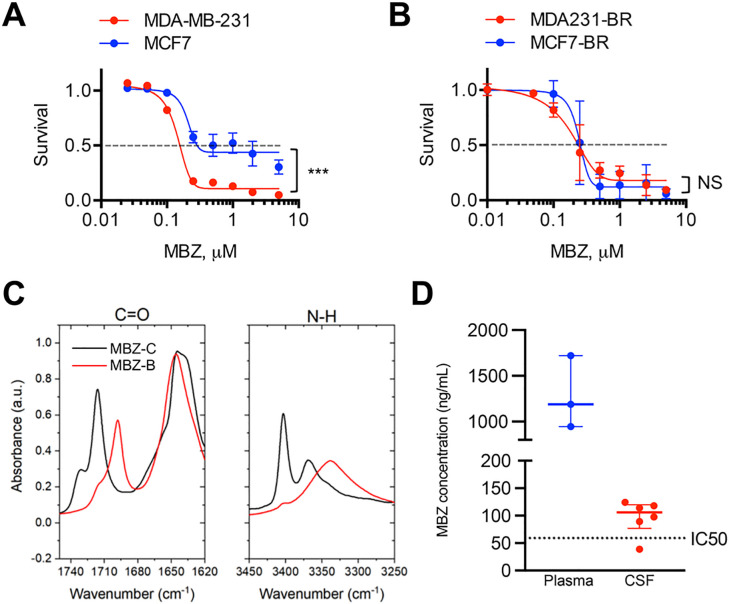
Mebendazole (MBZ) as a potential treatment against leptomeningeal disease. (A, B) Sensitivity to MBZ of TNBC cell line MDA-MB-231, hormone receptor positive cell line MCF7 (A), and their brain-tropic derivatives MDA-MB-231-BR and MCF7-BR (B). Brain-tropic MDA-MB-231-BR cell line is slightly more resistant to MBZ than the parental cell line MDA-MB-231: MDA-MB-231-BR (IC_50_=0.16 μM), MDA-MB-231 (IC_50_=0.14 μM), MCF7-BR (IC_50_=0.19 μM), and MCF7 (IC_50_ = 0.19 μM). (C) Infra-red spectra (FT-IR) of MBZ polymorphs revealing the presence of MBZ polymorph C (MBZ-C) and polymorph B (MBZ-B) in the MBZ from Sigma, CAS # 31431–39-7. (D) MBZ given at an oral dose of 100 mg/kg reaches therapeutic concentrations in the cerebrospinal fluid (CSF) of NuNu mice (median [MBZ] = 105.9 ng/ml ~ 0.36 μM). Plasma MBZ concentrations represent total quantity of MBZ, and CSF concentrations represent free, unbound MBZ. A horizontal line at 59 ng/mL corresponds to the IC_50_ = 0.20 μM of MBZ. MDA231 = MDA-MB-231. MDA231-BR = MDA-MB-231-BR. Significance: ***, p < 0.001, NS = not significant.

MBZ is one of the most common benzimidazoles commercially available and has been previously studied in a variety of cancer models. The MDA-MD-231 (IC_50_ = 0.14 μM) cell line exhibited higher sensitivity to MBZ compared to MCF7 (IC_50_ = 0.19 μM), which was consistent with the high-throughput screen results (Fig. 2A). Sensitivity to MBZ was not different between brain-tropic MCF7-BR and MDA-MB-231-BR cells (Fig. 2A,B).

MBZ is commonly manufactured as a mixture of several different polymorphs that are all variably bioavailable and differentially penetrant of the blood-brain-barrier [13]. FT-IR analysis of two different MBZ manufacturers revealed Polymorph C (in black) as the predominate polymorph in one sample (Sigma Aldrich Cat# M2523), as identified by the location of carbonyl and amine functional group absorbance (Fig. 2C). Polymorph B (in red) was the predominate polymorph in the sample from the second manufacturer (Sigma Aldrich Cat# 1375502). Since Polymorph C was previously described to have superior blood-brain-barrier penetrance than other polymorphs, we used MBZ from Sigma Aldrich Cat# M2523 for all subsequent experiments. CSF and plasma samples taken 4 hours after oral MBZ administration at a 100 mg/kg dose reached therapeutic concentrations (Fig. 2D), with an average CSF/plasma ratio of 0.09.

### MBZ reduced the migration of TNBC cell lines MDA-MB-231 and MDA-MB-231-BR

**Figure 3:**
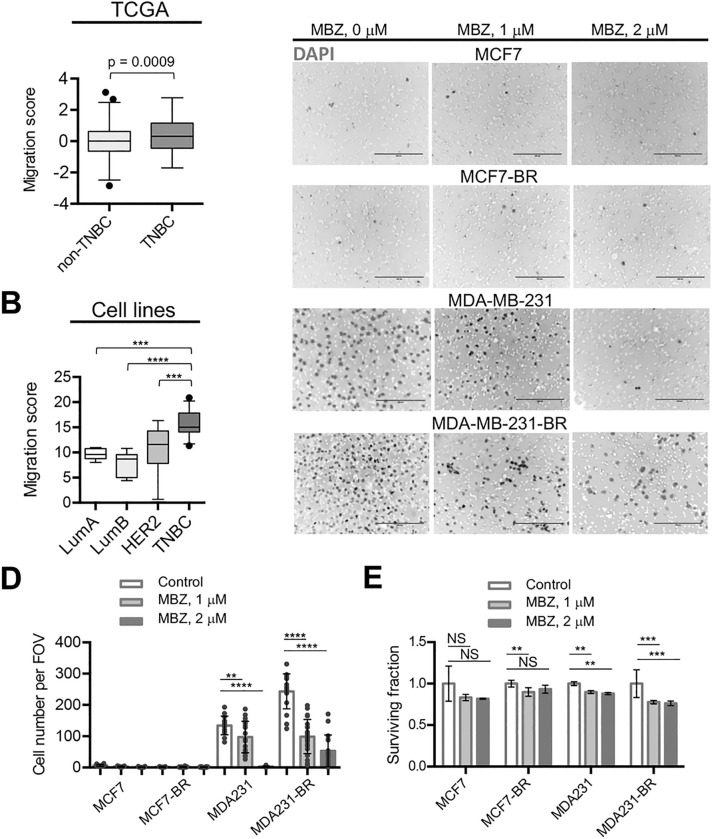
MBZ reduces the migration of TNBC MDA-MB-231 and MDA-MB-231-BR cells. (A, B) TNBCs have higher migratory capacity compared to other breast cancer subtypes. Migration scores from Nair et al. [7] were compared among breast cancer subtypes in TCGA patient data (A) and cell lines (B). (C, D) MDA-MB-231 and MDA-MB-231-BR have higher migration capabilities than MCF7 and MCF7-BR cells. Migratory capability of MDA-MB-231 cells increases upon acquiring brain-tropic status and is effectively inhibited by MBZ. Neither MCF-7 nor MCF-7-BR migrated significantly during 20 h. (C) Representative inverse fluorescence images of DAPI-stained membranes from Boyden chamber during 20 h. DAPI-stained cell nuclei are shown as dark gray spots in the background of white membrane pores. Scale bar: 200 μm. (D) Quantitation of migration in MCF7, MDA-MB-231, and brain-tropic MCF7-BR and MDA-MB-231-BR cells from (C). (E) Survival during 20 h treatment with MBZ. Plating efficiencies of untreated cell lines were not significantly different. MDA231 = MDA-MB-231, MDA231-BR = MDA-MB-231-BR. FOV = field of view. Significance analysis: ANOVA, **, p < 0.01, ***, p < 0.001, ****, p < 0.0001.

Like most benzimidazoles, the mechanism of action of MBZ hinges on selective binding to tubulin of helminths [27] (Table S1). We hypothesized above that benzimidazoles may be effective against migrating cancer cells in the same way they are effective against migrating cells in the developing nematode. *Cell ToPhenotype* predictor developed by Nair et al. [7] applied to both the TCGA patient tumor samples (Fig. 3A) and to breast cancer cell lines isolated from patient tumors (Fig. 3B) identified TNBCs as the most migratory cancers compared to other breast cancer subtypes. Supporting these results, we showed using *in vitro* migration assays that the triple-negative MDA-MB-231 cell line (migration score = 14.8 by the CellToPhenotype predictor [7]) exhibited notably greater migration capability than the luminal A MCF7 (migration score = 10.5) cell line (Fig. 3C,D). Importantly, the migratory capability of the MDA-MB-231 cells increased even further upon acquiring brain-tropic status, while the migration of both MCF7 and MCF7-BR remained low (Fig. 3C,D). During the 20-hour migration period MBZ effectively inhibited migration of both MDA-MB-231 and MDA-MB-231-BR cell lines (Fig. 3C,D), while cell survival was only modestly affected by MBZ in all cell lines (Fig. 3E). Nair et al. [7] have previously demonstrated that cytoskeletal drugs (such as tubulin binder MBZ) are more effective against cancers with high predicted migration capacity. Consistently, MBZ has been shown to slow down tumor growth and/or prevent metastatic spread of TNBC in animal models using multiple cell lines [14, 38, 39]. To our knowledge, no studies were comparing MBZ effect on tumor growth and/or survival for distinct breast cancer isotypes. Therefore, we decided to proceed with comparative testing of MBZ and hypothesized that MBZ would be more effective in the MDA-MB-231-BR-based LMD model compared to the MCF7-BR-based LMD model.

### MBZ effect in mouse model of TNBC LMD

**Figure 4:**
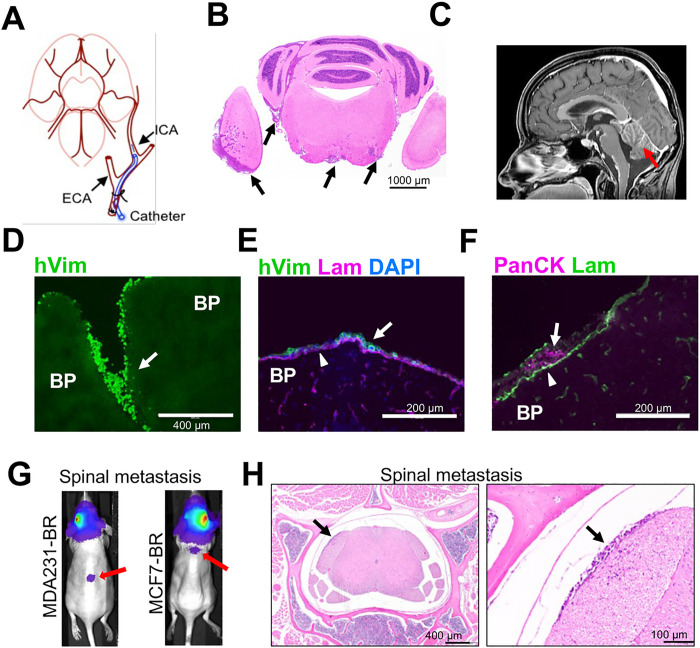
The model of leptomeningeal metastasis. (A) Schematics of an internal carotid artery injection of tumor cells to establish a murine model of LMD. (B) H&E-stained section from mouse brain affected by LMD. Arrows point to cancer cells in leptomeningeal space. (C) Patient brain T1W+C MRI sequence shows the anatomical location of leptomeningeal disease (LMD) (red arrow). (D-H) Immunofluorescence images depict dissemination of neoplastic cells into leptomeningeal space. BP = brain parenchyma. Arrows point to cancer cells in leptomeningeal space. (D, E) Vimentin, a marker of epithelial-to-mesenchymal transition, is highly expressed in MDA-MB-231 cells. Antibody against human vimentin (hVim) identifies MDA-MB-231-BR breast cancer cells. (F) Antibody against pan-cytokeratin (PanCK) identifies MCF7-BR breast cancer cells. (E, F) Antibody against laminin (Lam) shows the location of pia. (G) Bioluminescence images reveal intracranial disease and spinal dissemination (red arrows). (H) Spinal metastases identified by bioluminescence were verified by subsequent H&E staining. Right panel is a magnified version of a region indicated in the left panel. Black arrows point to the same spinal metastasis in the 4x image and a magnified (x20) image.

Previous studies suggested that migration, rather than proliferation, was predictive of breast cancer patient survival [7]. The stronger inhibitory effect of MBZ on migration of the triple-negative MDA-MB-231-BR versus luminal A MCF7-BR cells (Fig. 3C,D) implied that MBZ might be more effective against TNBC LMD. To produce LMD in animal models we injected cells using the ICA injection method (Fig. 4A), which we have previously shown to result in leptomeningeal spread [23] similar to human LMD (Fig. 4B,C). We chose the ICA method over the more widely used intra-cardiac method to produce CNS-specific metastasis clear of systemic spread which often accompanies the intra-cardiac injection.

To identify the MDA-MB-231-BR cells in mouse brain we used antibody against human vimentin (hVim), a marker of epithelial-to-mesenchymal transition that is highly expressed in the MDA-MB-231 cells [40] (Fig. 4D). MCF7-BR cells were identified by staining with antibodies against pan-cytokeratin (PanCK) or human estrogen receptor (ESR1). Both cell lines produced metastases in leptomeningeal space, as shown by the location of cells relative to the pia basement membrane identified by anti-laminin (Lam) antibody staining (Fig. 4E,F). The ICA injection of MDA-MB-231-BR cells resulted in predominantly leptomeningeal spread, although invasion into brain parenchyma at the late stages of disease progression was not uncommon (Fig. 4B, Fig. S2). Parenchymal involvement was different between the cell lines, with MCF7-BR cell line producing more globular and less invasive metastases than the MDA-MB-231-BR cell line (Fig. S2). Presence of parenchymal metastases is consistent with the clinical observations, where in up to 83% of LMD patients, leptomeningeal metastases coexist with parenchymal brain metastases [3, 4, 37]. LMD displayed similar gross heterogeneity in both models, where large bulky metastases coexisted with a population of single-cell spread/small metastatic clusters [23] (Fig. S3). Finally, as in human LMD, some animals developed spinal metastases at later stages (Fig. 4G,H).

**Figure 5:**
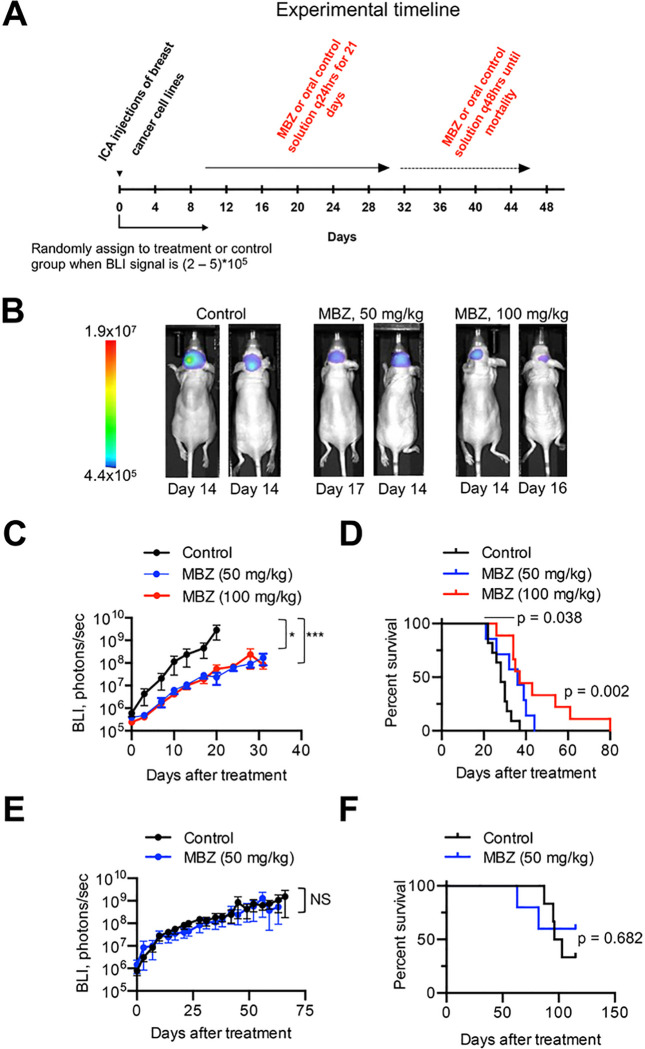
MBZ is effective against TNBC LMD in a mouse model. (A) Experimental timeline. (B) Representative bioluminescence images of mice with LMD in control and treatment groups (at 50 and 100 mg/kg). (C, D) MBZ slows metastatic growth as detected by bioluminescence imaging (C) and improves survival (D) in a mouse model of TNBC LMD formed by the injection of MDA-MB-231-BR cells into internal carotid artery of NuNu mice. (E, F) MBZ (50 mg/kg) shows no effect on metastatic growth (E) and survival (F) in the MCF7-BR model of LMD. Significance: *, p < 0.05, ***, p < 0.001, NS = not significant. Experiments in (C, E) were analyzed using repeated-measures method. Post hoc pairwise comparisons were performed using a Tukey adjustment for multiple comparisons.

The experimental timeline is shown in Fig. 5A. MBZ had a notable effect on the growth of MDA-MB-231-BR tumors at both 50 mg/kg and 100 mg/kg doses (Fig. 5B,C and Fig. S4). Little difference in mean BLI signal was observed between the 50 mg/kg and 100 mg/kg groups. Histological examination of brain sections revealed that MBZ effectively reduced single-cell metastatic dissemination, resulting in fewer new small metastases (Fig. S3B). Compared to the control, MBZ treatment significantly extended survival (Fig. 5D). No statistically significant difference in survival was observed between animal groups treated with 50 mg/kg and 100 mg/kg MBZ doses. In the MCF7-BR model, neither tumor growth (Fig. 5E) nor survival (Fig. 5F) were significantly affected by MBZ treatment (50 mg/kg). Animal weights were not significantly different between treated and control groups (Fig. S5 and Table S3), suggesting low toxicity.

## Discussion

LMD is a devastating diagnosis for patients with TNBC. Given the universally poor prognosis and limited efficacy of standard-of-care treatments, the investigation of alternative therapies for LMD carries increased importance. Drug repurposing confers obvious advantages in time-to-approval, cost, and safety. We identified MBZ as a drug that through its ability to hamper dissemination of highly migratory TNBC cells may, therefore, be a suitable candidate for LMD treatment.

MBZ was developed in the 1960s to treat a range of gastrointestinal helminth infections, and it is still one of the most commonly used medications in the world. MBZ safety has been evaluated in 6276 subjects in 39 clinical trials [18]; it can be taken safely in humans at doses as high as 200 mg/kg/day [18, 41, 42] and in rare cases, has been used in humans to treat CNS infections, including neurocysticercosis and echinococcus [17, 43, 44]. Indeed, its relatively small size and lipophilic properties render it an appropriate agent to be repurposed for CNS pathologies [10, 13, 20].

MBZ was successfully tested in multiple preclinical tumor models, including glioma [11, 13, 45] and TNBC [14, 38]. Several active and/or recruiting clinical trials investigating the anticancer effect of MBZ, alone or in combination with other drugs, are currently registered at clinicaltrials.gov [18, 19]. This includes the recent Phase I study conducted by Patil et al. exploring the safety of high-dose MBZ among patients with recurrent glioblastoma [19]. In 11 patients, no dose-limiting toxicity was reached, and the rate of adverse events was low, even when used in combination with temozolomide or lomustine. Other studies, including those involving high-grade glioma, are actively enrolling patients.

Our study is the first effort to test the efficacy of the drug in the treatment of CNS metastasis. We were able to demonstrate that the oral administration of MBZ, at both 50 mg/kg and 100 mg/kg doses, was able to slow tumor growth and increase survival in an aggressive preclinical model of TNBC LMD. Importantly, our dosing protocol, in which mice voluntarily consumed MBZ in a mix of sesame oil and honey, reached therapeutic concentrations in the CSF. The median CSF concentration was 106 ng/mL for animals treated at the 100 mg/kg dose, which was almost twice the IC_50_ of 56 ng/mL. While CSF concentrations at the 50 mg/kg dose were not measured, animals treated at that concentration still experienced slower tumor growth and increased median survival, both at statistically significant levels, suggesting a robust therapeutic effect.

Bai et al. [13] reported the variability in the efficacy of MBZ across different batches, emphasizing its dependence on the polymorph content. Furthermore, bioavailability and efficacy of MBZ are known to be influenced by intake of fat, which strongly facilitates benzimidazole absorption [46]. These findings suggest that these factors alone could introduce substantial variability in drug efficacy across studies and potentially impact the outcome of clinical trials. Therefore, the dependence of MBZ bioavailability on the drug administration protocol (particularly, fat content) and dosage formulation should be taken into account. For instance, in our study MBZ consisted of highly bioavailable polymorphs B and C, which are optimal for maximum efficacy. Yet, the plasma levels of MBZ in our study, albeit therapeutically significant, were notably lower than those reported by Bai et al. [13]. The observed disparity in plasma MBZ levels compared to the reference study [13] may have resulted from significantly lower fat uptake per MBZ dose in our case.

Our data suggest that MBZ targets cancers with high migratory capacity, and may be particularly effective when these cancers spread into leptomeningeal space, where cancer cell migration could be further enhanced in response to abundant cytokine/chemokine signaling [47]. We’ve shown that among breast cancer subtypes, the TNBCs had the highest migration potential. Consistent with the strong inhibitory effect of MBZ on migration of TNBC MDA-MB-231-BR cells, MBZ extended survival of TNBC LMD mice. The non-migratory luminal A MCF7-BR cells produced less aggressive LMD, and were non-responsive to MBZ both in *in vitro* migration assay and in *in vivo* LMD model.

## Conclusion

In summary, MBZ was demonstrated to be a safe and effective oral agent in an aggressive animal model of TNBC LMD. MBZ may function by selectively targeting migrating tumor cells. These findings are concordant with previous efforts involving MBZ and CNS pathology and further support the drug’s potential utility as an alternative therapeutic for TNBC LMD.
